# Advances in single-cell metabolomics to unravel cellular heterogeneity in plant biology

**DOI:** 10.1093/plphys/kiad357

**Published:** 2023-06-20

**Authors:** Kanchana Pandian, Minami Matsui, Thomas Hankemeier, Ahmed Ali, Emiko Okubo-Kurihara

**Affiliations:** Metabolomics and Analytics Centre, Leiden Academic Centre for Drug Research, Leiden University, Einstein Road 55, 2333 CC Leiden, The Netherlands; RIKEN, Center for Sustainable Resource Science, Kanagawa 230-0045, Japan; Metabolomics and Analytics Centre, Leiden Academic Centre for Drug Research, Leiden University, Einstein Road 55, 2333 CC Leiden, The Netherlands; Metabolomics and Analytics Centre, Leiden Academic Centre for Drug Research, Leiden University, Einstein Road 55, 2333 CC Leiden, The Netherlands; RIKEN, Center for Sustainable Resource Science, Kanagawa 230-0045, Japan; College of Science, Rikkyo University, Tokyo 171-8501, Japan

## Abstract

Single-cell metabolomics is a powerful tool that can reveal cellular heterogeneity and can elucidate the mechanisms of biological phenomena in detail. It is a promising approach in studying plants, especially when cellular heterogeneity has an impact on different biological processes. In addition, metabolomics, which can be regarded as a detailed phenotypic analysis, is expected to answer previously unrequited questions which will lead to expansion of crop production, increased understanding of resistance to diseases, and in other applications as well. In this review, we will introduce the flow of sample acquisition and single-cell techniques to facilitate the adoption of single-cell metabolomics. Furthermore, the applications of single-cell metabolomics will be summarized and reviewed.

**ADVANCES**

- Single-cell analysis is a technique that measures only the target cell itself and can extract information that would be buried in bulk-cell analysis with high-resolution.
- This is a powerful tool when dealing with phenomena where cellular heterogeneity plays an important part e.g. response to stress or drugs.
- Single-cell metabolomics is an analysis method that instantly captures the metabolic fingerprint corresponding to the exact state visible under a microscope, unlocking the potential for multimodal analyses and leading to a deeper understanding of biological phenomena.

## Introduction

Each cell is unique in its behavior and reacts distinctively to its environment. This heterogeneity impacts biological and pathological processes yet to be fully explored.

To address this, a myriad of single-cell analytical techniques has emerged that aims to gain deeper insights into cell characteristics and their response to perturbations by analyzing their genome, transcriptome, proteome, and metabolome (Altschuler and Wu 2010).

Similarly, plant science is also experiencing such impacts, and therefore, single-cell metabolomics (SCM) study encompasses the analysis and measurement of the metabolic profile of individual plant cells. It has various applications involved in different areas such as plant development, stress response, and disease resistance. Such analysis allows a deeper understanding of cellular metabolism in plants and provides insights into metabolic heterogeneity and dynamic changes in response to various stimuli. This information will deliver metabolic differences between individual plant cells which aids a better understanding of the underlying mechanisms of plant growth and adaptation (Misra et al. 2014; Katam et al. 2022).

Recent studies have shown that even in populations that are relatively homogenous, differences in the metabolism on the single-cell level due to heterogeneity or changes in microenvironment have several biological implications (Katam et al. 2022). Furthermore, SCM can measure the distribution of metabolites in 3D inside a single cell, which allows us to gain deeper understanding of molecular processes inside the cellular division (Okubo-Kurihara et al. 2022). Metabolite production and accumulation may be different between various cell types such that a large portion of the current metabolic knowledge is misleading. Finally, since changes in the microenvironment or external stimuli causes rapid alterations in the metabolome on the single-cell level, SCM is the only technique that can capture these dynamic changes over time, whereas bulk analysis provides only a snapshot of the average metabolite levels across the whole population (Heinemann and Zenobi 2011; Jindal et al. 2018; Ali et al. 2022; Lanekoff et al. 2022).

Studying the metabolome, i.e. metabolomics, has become the field of choice for the comprehensive understanding of cell cycles, cell perturbations, and general cell functions due to its ability to translate current cell behavior into biochemical terms.

In specific, tracer-based approach using stable isotopes can reveal which part of metabolic network are being utilized, and analyzing the concentration flux in a biological pathway is helpful to determine directional changes in metabolism (Samarah et al. 2020).

However, SCM is challenging due to the small sample size (single cells) and the inability to amplify the signal like in genomics or transcriptomics. The major advantage of metabolomics is that it provides a snapshot of the current physiological state of the plant. Transcriptomics and proteomics can provide information about which genes or proteins are being expressed in a particular tissue or under a particular condition, but metabolomics can reveal how these changes are actually affecting the plant's metabolism and physiology. Also, it can provide information about the downstream effects of changes in gene expression or protein activity.

In addition, metabolomics can reveal metabolites that have been perturbed due to an intervention of the drug. Furthermore, the concentration flux in a biological pathway is important to determine directional changes in metabolism. Stable isotope tracers can relatively easily reveal which parts of the metabolic network are being utilized, which is difficult to predict with proteomics or transcriptomics (de Souza et al. 2020; Guo et al. 2021).

In recent years, MS (mass spectrometry), coupled with miniaturized sampling and processing techniques, has emerged as key technology that enables the study of the metabolome at the single-cell level. In this review, the workflow of SCM using MS will be critically examined and reviewed from cell isolation and sample preparation to measurement. Furthermore, the utility and application of SCM in the field of plant biology will be highlighted, with specific attention being given to several advanced technologies on single-cell sampling and various cell-cycle metabolism studies.

## Single-cell sampling

Sampling is a crucial step for any SCM experiment. Challenges in single-cell sampling are often centered around the small cell size and volume available for analysis. This is exacerbated further in the case of metabolomics where the inability to amplify the metabolite signal, unlike DNA or RNA, and the sensitivity of the metabolome to changes in the microenvironment pose significant hurdles in single-cell sampling. These challenges and the method of sampling used impact downstream metabolic analysis and the resulting biological interpretation of said analysis. Therefore, careful consideration is needed when choosing the correct sampling method. Several techniques have been developed or adapted to sample and isolate single cells with these challenges in mind. They can be broadly classified into capillary sampling techniques and microfluidics-based methods. In the following section, both techniques, their uses, and limitations will be discussed and critically reviewed.

### Capillary sampling methods

Microcapillary sampling methods, where a single cell is sampled using a fabricated micro glass capillary with a fine tip of inner diameter based on the cell size, are well established. These methods require a microscope and a manipulator to localize and aspirate a single cell. The prominent advantage of this method is that sampling will be achieved in the cell's native environment. However, automation is a major problem resulting in low throughput sampling. Additional strategies have been applied to sample single cells for metabolic analysis as detailed below.

Single-cell sampling and analysis were performed by inserting a fine oil-filled glass microcapillary into the cell. The capillary was mounted on to a micromanipulator, and operations were viewed under a microscope. The cell sample was expelled from the capillary by increasing the pressure in the oil using a full cell-pressure probe setup. This sampling setup was coupled with X-ray microanalysis to determine the significant inorganic vacuolar constituents in individual wheat cells (Malone et al. 1989). Plant-specific mesophyll cells were also sampled by inserting the microcapillary through a stomatal pore (Koroleva et al. 1997; Koroleva et al. 1998). These techniques have been applied not only to metabolic readout but also to multiple analyses.

Another live cell-sampling approach for MS using a video-microscopic method has been developed. In this method, the cells, or its contents, are sucked out by a glass capillary and directly sprayed into the MS using electrospray ionization (ESI). However, this method lacks sensitivity, and additionally, single-cell molecular detection was not achieved (Masujima 2009). After further development, a live single-cell MS (LSC-MS) technique for untargeted metabolic analysis was extensively explained by Fujii et al. 2015 in which the sampled plant cell was sprayed directly on to the MS. The initial procedure involved sampling a single cell with a 3D micromanipulator fixed to a microscope. The sample dish was placed under the microscope to choose single cells for sampling. Whole cells or the cell contents were drawn out using a nano spray tip or a microcapillary tip (with a diameter based on the cell size) using a 3D manipulator attached to a syringe that was used to apply positive and negative pressure to pull out the samples. Ionization solvent was injected using a pipette into the other end of the microcapillary. Finally, this nano spray tip was placed against the MS inlet, and a high voltage was applied to introduce the contents into the MS. As a result, many metabolites signals were obtained for further analysis (Fujii et al. 2015). Using the same sampling technique, the metabolic profile of plant cell mitosis was analyzed for its dynamicity (Okubo-Kurihara et al. 2022).

The major advantage of these capillary techniques on single-cell sampling is that sampling occurs with minimal disruption to the cells’ microenvironment and therefore reduces internal biochemical changes in the cell during sampling (Fujii et al. 2015). However, there are major drawbacks, including a longer sampling time and getting the correct microcapillary diameter. The latter depends on the cell type and cannot be more than 6 microns or the spray to the MS will be ineffective when the voltage is applied. In addition, there is a chance that the cells will stick to the capillary edge if the capillary is not positioned correctly, and there may be sample evaporation within the capillary if there is a delay in measurement. A further major complication with this technique is that it is not possible to retrieve the sampled cells from the capillary for culture. To circumvent these low throughputs and cell integrity issues, microfluidics-based sampling procedures have been developed as described in the following methods.

### Microfluidics-based single-cell sampling/sorting

Microfluidics-based approaches are uniquely suited to SCM due to their ability to handle small volume samples and have operations with limited dilution and high throughput. Microfluidics techniques used in single-cell analysis can be broadly classified into label-free and labeled methods. These include droplet-based microfluidics and acoustofluidics (label free) and fluorescence sorting (labeled) methods. In this section, these technologies will be discussed, along with some of their applications in SCM of plant cells.

Droplet microfluidics is a promising technique that is suitable for single-cell sampling and sorting from a bulk cell population (Autebert et al. 2012; Mazutis et al. 2013; Wang et al. 2014). An advantage of it is the rapid mixing inside the droplets, which aids rapid reactions if cells are treated with a reagent (Teh et al. 2008). Single-cell sorting using droplet microfluidics has been performed mostly in eukaryotic (Gach et al. 2016; Terekhov et al. 2017) and bacterial cells (Martin et al. 2003; Huebner et al. 2007). Fewer single-cell experiments have been done in plant genomics and transcriptomics with the possibility of amplification and increase the protein concentrations (Klein et al. 2015; Macosko et al. 2015; Yu et al. 2018). In a typical microfluidic setup, various parameters for droplet generation need to be kept in mind, such as chip material, size of the orifices, fluid viscosity and density, and capillary number. All these properties were considered in the study of Yu et al.'s group where they performed a droplet-based screening of single plant cells. In this work, they sorted plant protoplasts encapsulated individually in aqueous microdroplets based on the genetic expression of a fluorescent protein. On-chip encapsulation and analysis of protoplasts isolated from *Marchantia polymarpha* using a microfluidic system were carried out to quantify the stochastic properties of a promoter across a transgenic protoplast population and analyze the gene expression actively in response to environmental conditions. Droplet-based isolation of the protoplasts expressing the yellow fluorescent protein from mature thalli (wild type) was achieved by automatically sorting them using dielectrophoretic force (Yu et al. 2018). Droplets were sorted based on their fluorescence and cell specificity using microfluidic-based fluorescence-activated droplet sorting. Benefits of this technique include minimal reagent volumes (12 *µ*L of aqueous phase generates 10^6^ droplets), short setup time at below 10 min per sample, and high enrichment. Furthermore, additional development involving the integration of the sorting device with modules for droplet splitting will generate high-speed manipulations (Baret et al. 2009; Utharala et al. 2018). Droplet sorting can not only be achieved based on fluorescent properties but also on droplet size (Huh et al. 2007; Tan et al. 2008; Li et al. 2018), deformation (Chang et al. 2020), density (Deshpande et al. 2017) and directly using noninertial lift-induced forces (Hazra et al. 2019), sheath fluid properties with acoustics (Karthick et al. 2018), and mechanical properties (Sajeesh et al. 2014). Fluorescent tags may change the metabolome, and hence sorting by a cell's physiological and mechanical properties is highly efficient, especially for further downstream analysis (Jayaprakash and Sen 2019). As the technology has advanced further, droplets have been sorted using an automated system that performs real-time dual-camera imaging. The objective of this system is to replace manual cell handling techniques with automation via machine learning algorithms (Tan et al. 2008).

Major advantages of droplet-based single-cell sorting are sample handling with minimal dilution, low carry over, and high throughput. Major disadvantages are that the device fabrication is complicated depending on the different sample treatment steps and the droplet varies with cell size. However, ESI produces charged ions directly from the charged liquid, so it is ideal for coupling with the microfluidic platform.

To achieve an oil-free sorting mechanism, acoustic-based sorting of cells and detection in MS is an ideal procedure. Acoustic-based single-cell sampling is an interesting pipeline for metabolic analysis due to its well-known advantages. Acoustic or sound energy is nothing but kinetic mechanical energy, a result of when sound or pressure makes a substance or cells vibrate. This energy moves through the substance/cells in waves. Through these vibrations, particles tend to move according to their mechanical and sound characteristics and at a different range of wavelengths. Acoustic energy distribution in microfluidics technology enables the use of minute volumes of fluid for analysis, and hence, it is ideally suitable for single-cell sorting from bulk cell numbers (Wu et al. 2019). This method also highlights that sorting can be achieved label free without destroying the cells which can then be grown on in culture after the isolation (Karthick et al. 2018; Zhou et al. 2019). An advanced acoustofluidics development is acoustofluidic fluorescence-activated cell sorting (FACS) that has high-throughput, high-resolution cell detection, and sorting in a single chip. This device has sorted labeled cervical cancer cells and polystyrene beads from nonlabeled ones (Nawaz et al. 2015). Major advantages of acoustic-based cell sorting are high throughput, ease of incorporation into microfluidics, high purity, biocompatibility, maintaining high integrity of the cells, and label-free sorting. Major limitations include complicated device fabrication procedures and oversensitivity to fluid properties. To overcome these, cells can be tagged with specific antibodies and sorted in a high-throughput manner. This fluorescence-activated cell sorting technique is highly effective and is discussed in the section below.

### FACS/sampling

Flow cytometry or FACS is equipped with a fluid system where the sheath fluid sends the cell suspension solution in a uniform stream flow. The fluid flow in a microfluidic approach is very much controlled by a tunable flow rate to focus the cell content into the MS (Li et al. 2021). In plants, cell-specific analysis of Arabidopsis leaves has been done using laser-capture microdissection (LCM) or GFP (green fluorescent protein)-expressing plants used for protoplast generation and subsequent FACS, the biotinylated nuclei using BLRP (biotin ligase recognition peptide) for nuclear precipitation and immunoprecipitation of polysomes (Grønlund et al. 2012). In addition, maize protoplasts sorted using FACS were efficient at generating protoplasts from root and shoot inner layers, and FACS has also been applied to *Arabidopsis thaliana* at the single-cell level to isolate protoplasts for tissue-specific transcriptome profiling (Sheen 2001; Warnasooriya and Montgomery 2010; Sparks and Benfey 2017; Ortiz-Ramírez et al. 2018; Satterlee and Scanlon 2022).

## Single-cell MS

Many methods have been developed to deal with the unique challenges in SCM (Pan et al. 2014; Nakashima et al. 2016; Zhu et al. 2017; Hansen and Lee 2018).

These methods have been used in different metabolic studies investigating organic acids (Gomez-Zepeda et al. 2021), lipids (Harkewicz and Dennis 2011), and microbial metabolism on the single-cell level (Gao and Xu 2015). MS-based single-cell metabolomic techniques can be classified into microsampling, microfluidics, and MS imaging methods.

In this section, the advances in these techniques will be critically reviewed in terms of their advantages and limitations, as well as their compatibility with different single-cell experiments.

### Single-cell microcapillary sampling and MS integration

Microsampling techniques aim to combine microscopy data with MS measurements with minimal disruption to the cell's microenvironment. This is often achieved by means of a pulled glass capillary or probe that is manipulated using a 3D micromanipulator attached to a microscope. The probe is then used to sample a whole cell, or in some cases, parts of the cell. Afterwards, the sample is then transferred to the MS instrument of choice for analysis.

LSC-MS is a microsampling technique that utilizes a metal-coated glass capillary to sample cells and then introduces them to the MS via nESI (nano-electro spray ionization) technique. The method is already described in the previous sampling section. By using the same capillary for sampling and measurements, sub-attomolar sensitivities can be achieved (Fujii et al. 2015). LSC-MS method is unique as it links information from microscopy with MS data. This facilitates a cell-specific analysis in a label-free condition and unlocks more insights about the cell behavior. With this method, Okubo-Kurihara et al.’s group examined the metabolic profile of tobacco cell's each mitotic subphase, prophase, metaphase, anaphase, and telophase, with high sensitivity and selectivity. The study revealed the metabolic differences between the 4 mitotic subphases and leads to a high molecular weight lipid accumulation at prometaphase by inhibiting microtubules polymerization. This proved that there is no direct association of microtubule structure with cellular metabolism at single-cell level (Okubo-Kurihara et al. 2022).

To achieve more precision in sampling volume of single cells, electromigration can be used to sample and introduce cells to the MS. In this method, single yeast cells were sampled using a microcapillary and migrated to the capillary tip using controllable electro migration by a direct current of 1.2 kV, lysed with a brief time pulses and then driven to nESI-MS. However, this technique requires optimization of electro migration and electroporation voltages for each cell type because the arrangement of the cell wall layers that comprise cellulose, microfibrils, hemicellulose, pectin, lignin, and soluble proteins varies between cells. Therefore, achieving electrical lysis or to migrate a cell to the capillary tip different potential is needed depending on the cell wall composition (Olefirowicz and Ewing 1991; Li et al. 2020). Some notable limitations of these methods are it cannot be used for on-line and dynamic monitoring of the metabolome of single cells since in both techniques, the sampling of the cells is done either offline (LSC-MS), or whole cells are sampled which prohibits repeated measurements.

This limitation was addressed by Yang et al.'s group and the development of the T-probe. The T-probe can continuously sample the cell of interest through a glass pipette with simultaneous injection of an extraction and ionization solvent which introduces the sample to the MS using nESI-MS via a T-piece connection (Liu et al. 2018; Zhu et al. 2019). The T-probe was used for quantitative analysis of abscisic acid (ABA) and jasmonoyl-L-isoleucine (JA-Ile) plant hormones at single-cell level. This study examined the endogenous ABA level which was compared using Triple Quad 5500 and Orbitrap Velos Pro with nano-ESI tips. The results demonstrated that the accumulation of ABA in dehydrated leaves was much higher than the intact leaves, and JA-Ile level was higher in wounded leaves than the unwounded ones (Shimizu et al. 2015). However, this method requires computational simulations for individual probes due to the variance in volume mixing i.e. sample and solvent volumes. A limitation that is also shared with other microsampling methods is the lack of separation techniques such as capillary electrophoresis (CE), ion mobility separations which can enhance the identification, and maximum coverage of metabolites.

Microsampling techniques are among the most sensitive single-cell metabolomic methods, especially when little to no dilution is applied to the sample prior to analysis. They also sample the cell with minimal perturbations to the microenvironment and therefore the metabolome. However, they also share the common limitation of having low throughput, due to the often manual and labor-intensive sampling process. Microfluidic-based MS methods attempt to address this limitation through high-throughput and automatable single-cell isolating and sorting followed by MS measurements.

### Microfluidics-based sampling MS integration

To achieve high-throughput sampling, microfluidics platforms, such as droplet microfluidics, acoustofluidics, and microfluidic-based flow cytometry, can be coupled to MS for high throughput detection of plant cell metabolites. Kempa et al. group showed that the potential of segmented flow, droplet microfluidics, and integration with the MS in single-cell analysis.

In this method, droplets were generated using egg white solution and the segmented oil phase reached the outlet of the emitter as plugs of that phase and interdroplet spacing was maintained during MS measurement. The droplets generated in a microfluidic chip were connected to a stainless-steel emitter which was inserted through the rear of Nano source tip holder and secured in place using a stainless-steel nut. This setup was coupled to multiple ion sources such as drift tube ion mobility spectrometry (DTIMS) quadrapole times of flight (Q-TOF), traveling wave ion mobility spectrometry (TWIMS) Q-TOF, and Orbitrap coupling. The droplet frequency and the diameter were calculated using optical analysis (Kempa et al. 2020). However, electrospray instabilities were identified with the increased salt concentrations at the emitter outlet in droplet mode but not in direct infusion. Like this study, yeast cell's phytase enzyme was analyzed at single-cell level in nanoliter droplet volumes. This method works by transferring the droplets with yeast cells on a glass slide with custom-made hydrophilic/hydrophobic patterns to avoid the droplet spreading. With these techniques the method delivered the quantification of phytase secretion by a single cell and characterized the reaction of secreted enzyme with MALDI-MS to monitor the enzymatic reactions and distinguish various inositol phosphates even in the medium. This study needs further developments on droplet volume by avoiding wetting issues to achieve a better accumulation of enzyme (Haidas et al. 2019). To standardize the generated droplet volume, Smith et al.'s group achieved droplet stabilization using surfactants and used it in the analysis of cocktail droplets that contains cytochrome C, alpha-chymotrypsinogen A, carbonic anhydrase, and chicken lysozyme. However, cross-contamination was between residual proteins and the surfactant ions due to incomplete spraying of the sample before the arrival of the next droplet remains a challenge (Smith et al. 2013).

The aforementioned technologies can be used for single-cell plant metabolomics in principle (Fig.). The proof of that, Yu et al. 2018 analyzed sorting of plant protoplasts encapsulated individually in aqueous microdroplets, based on the genetic expression of a fluorescent reporter protein (Fig. A). These sorted individual protoplasts could be directed to the MS ion source to analyze its metabolites in a high-throughput manner with an idea of Kempa et al. 2020 model (Fig. B and C).

**Figure.**
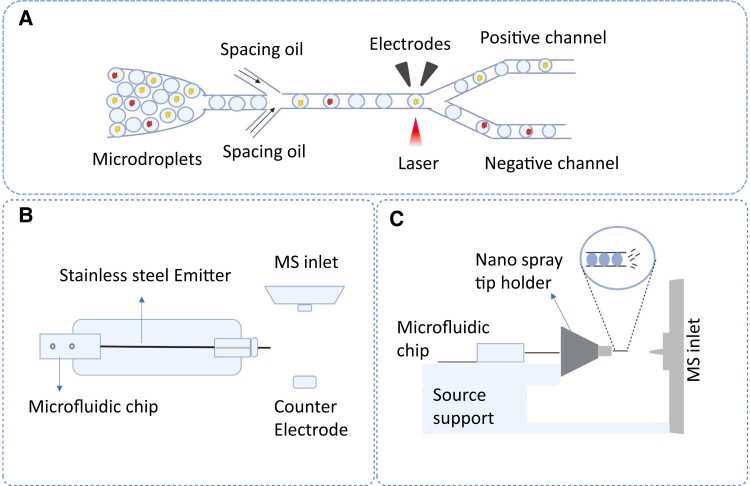
Illustration of integrating a microcapillary single-cell (SC) sampling method to droplet microfluidics for SC metabolomics studies. **A)** Illustration of a platform for droplet microfluidic sorting of encapsulated protoplasts based on the genetic expression of a fluorescent protein. **B)** and **C)** Top and side view of the Nanospray ESI source to incorporate microfluidic chip. (Figures are adapted from Yu et al. 2018; Kempa et al. 2020, and created with BioRender.com, accessed on FX2509SD0O, February 13, 2023.)

Evidently, the control on droplet size and stability can be easily managed due to broader range of plant cell sizes. However, considering the general limitations of droplet microfluidics like droplet coalescence, break down, and chip surface wetting, furthermore developments should be achieved in designing a chip with controllable and consistent droplet generation, high throughput, and the possibility for automation. Furthermore, microfluidic techniques and microsampling techniques cannot provide spatial information such as the distribution of a certain metabolite in the cellular space. One of the techniques that can provide such information on the single-cell level is MS imaging, which will be discussed in the upcoming section.

## Single-cell MS imaging

MS imaging methods were developed to provide biochemical information of biological models in 2D/3D. Furthermore, additional modalities such as dual microscopy/MS instruments can be incorporated to obtain both structural and chemical information of individual cells in 3D. Using this technique, *Allium cepa* epidermal cells were analyzed to identify the species-specific cell wall component as trisaccharide from several of its types using optical microscopy and laser ablation ESI MS (LAESI MS), (Taylor et al. 2021). The notable advantage of LAESI MS method is it offers alternatives for direct ionization of samples with minimal treatment than other traditional MS. However, this optical microscope is not suitable and efficient to analyze subcellular components microcompounds present in the sample due to lack of resolution. To achieve the highest sub cellular resolution, secondary ion MS (SIMS) is often used. SIMS is the prevailing technique used to map the distribution of endogenous biomolecules with sub-cellular resolution. It allows 2D and 3D localization of analytes in a variety of cells. SIMS is a label-free, matrix-free technique that utilizes a focused primary ion beam to desorb and ionize analyte molecules. The benefits of using SIMS are high spatial resolution and the ability to analyze molecules on the surface of biological cells like cell membrane components, and it is an ideal technique for single-cell analysis due to its high-resolution sensitivity in 2D and 3D level and sub-micron resolution (Passarelli and Ewing 2013). Although, sample ionization under a high vacuum may cause some fragmentation of some cellular components and affect structural analysis and detection sensitivity (Senoner and Unger 2012). Such limitations have been partially addressed by methods such as cryogenic orbiSIMS. This approach is operated under –100 °C to lower the vapor pressures of semi volatile organic compounds. This was used successfully to analyze semi-volatile organic compounds, and it limited vaporization prior to ionization (Newell et al. 2020).

One major limitation of SIMS technique is their low throughput. MALDI-MS is one of the main techniques used when higher throughput is needed in single-cell MS-imaging studies. MALDI is one of the main label-free techniques to visualize and study large molecules, e.g. drugs, lipids, and proteins, in single cells with minimal fragmentation and multiple-compound analysis in 2D and 3D molecular distributions (Xiong et al. 2016). It allows the samples to be mixed uniformly in a large quantity of matrix. The matrix absorbs ultraviolet light (nitrogen laser light −337 nm) and converts it to heat energy. Using MALDI-MS the rhizome of *Glycyrrhiza glabra* (licorice) where the localization of secondary metabolites such as flavonoids, flavonoid glycosides, and saponins was detected with 10 *µ*m spatial resolution and then correlated with the histological and metabolic features of licorice (Li et al. 2014). To achieve higher spatial resolution, modifications to the laser spot shape are often necessary. Takahashi et al. 2015 succeeded in mapping the 2D distribution of small metabolites in *A. thaliana* with high spatial resolution. This was achieved by the modifications of laser spot's shape and position and the combination of the shorter pulse width laser. With this new design, nontargeted analysis of small metabolites in *A. thaliana* was achieved in high resolution and high mass accuracy in 2D (Takahashi et al. 2015). Another instrument modified technique was identified by Korte et al. in which high mass resolution (5 micron level) was achieved by modifying beam-delivery optics of a commercial MALDI-laser ion trap-Orbitrap instrument, incorporating an external Nd:YAG laser, beam-shaping optics, and an aspheric focusing lens to reduce minimum laser spot size. With these modifications, maize leaf was sampled and distinct metabolites such as flavonoids (luteilin/kaempferol and rutin) from epidermal cells and chloroplast membrane lipids were measured in high resolution (Korte et al. 2015). Despite their utility, typical MS methods often lack separation steps or structure elucidation mechanisms due to their inherent design limitations. Integrating and combining other MS-based platforms can alleviate this issue. An example of this is an integrated experiment for comprehensive analysis of plant roots, where liquid chromatography MS (LC-MS) was used to elucidate the lipid profiles of 3 zones of seminal roots; inductively coupled plasma MS was used to study the elemental content of roots, and MALDI-MS was used to obtain spatial localization of the sample. This integrated system succeeded in analyzing and mapping ionic and metabolic response to salinity stress in plant roots and mechanisms to resist stress and stress responses were elucidated (Sarabia et al. 2018). Another drawback of MALDI-based methods is that the matrix used often interferes with measurements of low molecular weight ions. This drastically reduces metabolic coverage since most metabolites have low molecular weight. One such effort to address this was led by Brown et al., where new matrix enhanced surfaces were developed that were specifically optimized for low molecular weight compounds (Brown et al. 2015).

Desorption ionization electrospray MS (DESI MS) is another technique that can be analyzed in low molecular weight compounds with the added benefit of on-line coupling with HPLC for metabolite profiling. DESI-MS produces less fragmentation and is ideal for imaging low-quality regions with nondestructive and high throughput capacity (Costa and Graham Cooks 2008; Gao et al. 2022). Direct analysis of chlorophyll degradation products and their concentration on senescent tree leaf surfaces were analyzed successfully in a high-throughput manner using DESI-MS. In addition, the sensitivity of this analysis was enhanced by the introduction of an imprinting process using porous polymer material as substrate. This helped in evaluating more secondary metabolites present relatively in lower concentrations in the leaf matrices (Müller et al. 2011).

Single-cell MS imaging methods are mainly used to visualize the spatial distribution of biomolecules in 3D space with varying resolution. However, it also introduces the most perturbations to the cells’ microenvironment due to the often-extensive sample preparation involved prior to analysis. In addition, one of the major technical hurdles reported in SCM is the broad dynamic range of concentrations that can have a considerable impact on the observable metabolome, something that MSI methods are poorly equipped to handle, due to the lack of a separation or enrichment steps. Despite these challenges, MSI and the other techniques discussed before are already being used to uncover previously unknown insights about plant cell behavior on the single-cell level. Some of the applications of these technologies will be discussed in the following section. The summary of all available methods on single-cell sampling, and their detection techniques are compiled below in Table 1.

**Table 1.** Summary of references on advanced single-cell sampling methods and metabolites detection using different MS

Topics	Method	Advantages	Limitations	References
Single-cell sample acquisition	Live single-cell MS using capillary microsampling	Minimal disruptions to the cells microenvironment	Lack of high-throughput No possibility of sample preparation	Lanekoff et al. 2022 Malone et al. 1989 Koroleva et al. 1997, 1998 Masujima 2009 Fujii et al. 2015 Okubo-Kurihara et al. 2022
Microfluidics-based single-cell sampling techniques	Droplet microfluidics	High-throughput Automation Controlled volume Free of contamination	Relatively low sensitivity and robustness Complicated device fabrication techniques Large disruption to the cell content due to extensive manipulation	Mazutis et al. 2013 Autebert et al. 2012 Wang et al. 2014 Teh et al. 2008 Gach et al. 2016 Terekhov et al. 2017 Huebner et al. 2007 Martin et al. 2003 Yu et al. 2018 Macosko et al. 2015 Klein et al. 2015 Baret et al. 2009 Utharala et al. 2018 Tan et al. 2008 Huh et al. 2007 Li et al. 2018 Chang et al. 2020 Deshpande et al. 2017 Hazra et al. 2019
	Acoustic-based sorting and mechanical sorting	Label-free isolation High-throughput	Complicated device fabrication procedure No sample preparation Sensitive to fluid properties	Karthick et al. 2018 Sajeesh et al. 2014 Jayaprakash and Sen 2019 Wu et al. 2019 Zhou et al. 2019 Nawaz et al. 2015
	FACS	Highly selective sorting and High throughput	Labeled-sorting, chances of missing cells subpopulation due to lack of specific tags Large disruption to the cells metabolome due to extensive manipulation	Li et al. 2021 Grønlund et al. 2012 Ortiz-Ramírez et al. 2018 Satterlee and Scanlon 2022 Sparks and Benfey 2017 Sheen 2001 Warnasooriya and Montgomery 2010
MS for single-cell analysis	Ion-mobility triple/MS and nanospray ESI	Increased selectivity	Complicated data analysis	Fujii et al. 2015 Li et al. 2020 Olefirowicz and Ewing 1991 Liu et al. 2018 Zhu et al. 2019 Shimizu et al. 2015 Okubo-Kurihara et al. 2022
	21T FTICR-MS	High resolution Can resolve fine isotopic structures	Low throughput Complex instrumentation	Samarah et al. 2020
	DTIMS Q-TOF, TWIMS Q-TOF and orbitrap	High-quality spectra obtained from cell encapsulated droplets	Cross-contamination with some residual proteins to the surfactant ions due to incomplete spraying.	Kempa et al. 2020 Haidas et al. 2019 Smith et al. 2013
	LAESI-MS with ion mobility separation and LIAD	Less disruptions to cells microenvironment Higher selectivity than direct infusion-based methods	Cell media dependent Large spot size	Taylor et al. 2021
MS imaging	SIMS	High spatial resolution	Higher fragmentation ionization method	Passarelli and Ewing 2013 Senoner and Unger 2012
	MALDI-MS DESI-MS	Label-free technique Minimal fragmentation, multiple compound analysis in 2D and 3D molecular distributions	Lack of sensitivity Require additional derivatization reactions	Hansen and Lee 2018 Xiong et al. 2016 Li et al. 2014 Takahashi et al. 2015 Korte et al. 2015 Sarabia et al. 2018 Brown et al. 2015 Gao et al. 2022 Costa and Graham 2008 Müller et al. 2011

## SCM in plant biology

One of the most direct depictions of the phenotype would be to reveal the metabolomic information that makes up the observed object or the phenomenon itself (Schauer and Fernie 2006). In this manuscript, we described with a focus on common single-cell sampling and SCM methods regardless of the organism. In plants, technology has also progressed over the past decade, and detailed information is now available (Oikawa and Saito 2012; Katam et al. 2022). This section will show how these methods have been incorporated into plant research to reveal biological phenomena (Table 2).

**Table 2.** Summary of references on plant single-cell metabolome studies

Materials	Cell type	Sampling	MS	Metabolites	References
*Pelargonium zonale*	Leaf, stem, petal cell	Micromanipulator Nano spray tip	Nano-ESI-MS	Targeted (geranic acid, caffeine, and geraniol)	Lorenzo Tejedor et al. 2009 Masuda et al. 2018
*P. zonale*	Leaf, stem, petal cell	Micromanipulator Gold-coated glass capillary	Nano-ESI-MS	Nontargeted (m/z 100 to 1,000, teropenoids, isoprenoids)	Lorenzo Tejedor et al. 2012
*A. thaliana*	Epidermal cell (pavement, trichome, vasal cells)	Leaf hair depilation	GC-TOF-MS	117 primary metabolites	Ebert et al. 2010
*A. thaliana*	Epidermal cell (pavement, trichome, vasal cells)	Micromanipulator microcapillary	ESI-MS and IMS	Nontargeted (23 metabolites and lipids)	Zhang et al. 2014
*A. thaliana*	Root (Endodermis, epidermis, columella, cortex, and stele)	FACS	UPLC-qTOF-MS	Nontargeted (50 metabolites, glucosinolates, phenylpropanoids, dipeptides)	Moussaieff et al. 2013
*Solanum lycopersicum*	Trichome (stromal and glandular cells)	Micromanipulator Microcapillary	IEC-PPESI-MS	Nontargeted (flavonoids, acyl sugars)	Nakashima et al. 2016
*Catharanthus roseus*	Stem (idioblast and laticifer cells) Leaf (epidermal cells)	Cross section with microtome Syringe and nano-electrospray tip	Imaging MS Single-cell MS	Targeted (TIA precursors and TIAs)	Yamamoto et al. 2022 Yamamoto et al. 2019
*Cannabis sativa*	Trichome (stalked, sessile and bulbous)	Microcapillary	LC-MS	Targeted (cannabinoids)	Livingston et al. 2020
*Torenia hybrida*	Petal cell	Laser microsampling	LC-ESIMS	Targeted (anthocyanin)	Kajiyama et al. 2006
*Viola cornuta*	Petal cell	Directly placed and measured	MALDI TOF-MS	Targeted (flavonol 3-O-glycosides, violanin)	Sugahara et al. 2019
*Allium cepa*	Epidermal cell	Directly placed and measured	LAESI-MS	Targeted (cyanidin)	Shrestha et al. 2011
*Miscanthus* (*Miscanthus x giganteus*)	Dried and frozen sections	Raman microscopy and SIMS	Raman microscopy and SIMS, LDI-MS	Targeted (lignin, cellulose)	Li et al. 2010 Leng et al. 2022
BY-2 cultured cells	Mitotic phase cell	Micromanipulator Nano spray tip	Orbitrap LC-MS	Nontargeted (high molecular weight metabolites)	Okubo-Kurihara et al. 2022
*Celtis australis*	Giant internodal cell (cytoplasm and vacuole)	Cutting and content extraction	CE-MS	Nontargeted 125 metabolites	Oikawa et al. 2011
*Gossypium hirsutum* *A. thaliana*	Embryo, root seed, leaves <Lipid droplet>	Nanomanipulator Platinum-coated tip	Direct organelle MS (DOMS)	Targeted TAG	Horn et al. 2011 Horn and Chapman 2011
*Vicia fava*	Leaf cell	Micromanipulator Nano electrophoresis tip	LC-MS/MS	Targeted ABA, JA-Ile	Shimizu et al. 2015
*Oryza sativa*	Pollen	Piezo-manipulator Quartz capillary	picoPPESI-MS	Targeted Phosphatidylinositol	Wada et al. 2020

Plant metabolites are estimated to number up to 200,000, and the structure of plant cells differs from animal cells in that they have vacuoles, chloroplasts, and cell walls, but a variety of insights can be gained through a successful combination of targeted sampling, metabolite detection equipment, and databases (Lisec et al. 2006; Misra et al. 2014; Patel et al. 2021).

Single-cell analysis in plants is now often performed by microscopy and in situ imaging, which allows the isolation of cells that were mixed in large-scale analyses, by obtaining information corresponding to the phenotype of the cell under the microscope. This is thought to have the advantage of extracting buried metabolites and enabling more detailed analysis of biological phenomena (Zenobi 2013). Conversely, there must be information that can only be revealed by single-cell analysis. It is also necessary to take a broader view, bearing in mind that SCM plays a significant role in linking omics information between the phenome, genome, and transcriptome and facilitating functional inference (Hall et al. 2002; Saito et al. 2008).

## SCM in different plant tissues

Tissue-specific characteristics are revealed by differences in metabolite profiles.

One of the main analyses of SCM in plants is that of the metabolites in single cells from different tissues. The advantage of metabolomics (or proteomics) is the possibility to select factors that may explain the phenotype of interest without known information. Metabolomics is powerful because it can simultaneously analyze the biomolecules of a single cell, where gene expression (transcriptome) information affects a complex network of biomolecules, resulting in the phenotype that we see as the result. The significance of the metabolome is that it can find small molecule factors and quantitate them simultaneously, and by using imaging MS, it is possible to visualize the molecular distribution of various biomolecules, which provides information on biomolecules at once. In addition, comparison of transcriptome and metabolome and other omics information at the single-cell level should be more compatible. Metabolomics at the single-cell level has the potential to better define the boundaries that have distinguished different tissues that have been roughly divided as bulk.

A method has been developed for the rapid analysis of live single plant cells from different tissues, where the intact cells are observed by video microscopy, directly sampled on nano electrospray tips and analyzed using MS (nano-ESI-MS) (Lorenzo Tejedor et al. 2009; Masuda et al. 2018). MS analysis of metabolites in leaf cells and leaf stalk cells of living plants was successfully used to identify specific target substances such as geranic acid, caffeine, and geraniol. It has shown that it is possible to compare metabolites in distinct types of cells from different tissues while obtaining phenotypic information.

Further expanding on the research, live single cells of *Pelargonium zonale (L.)* leaf, stem, and petal tissue were directly inserted into a nano electrospray tip under the microscope, and metabolites in the m/z range of 100 to 1,000 were comprehensively detected by nontargeted high-resolution ESI-MS analysis. More than 1,000 metabolite peaks were detected even in samples as small as 1 to 5 pL. PCA analysis of leaf, stem, and petal cells showed that cells were clustered close to each other; hence, the characteristics of each tissue were extracted (Lorenzo Tejedor et al. 2009). While geranic acid was specific to leaves, whereas methyl citronellate was mainly was found in both leaves and stems, but rarely in petals. This information can be valuable in comparing different cells. However, further analysis is needed to clarify the metabolites and metabolic pathways that contribute to this tissue-specific metabolic profile and how they vary from tissue to tissue.

Untargeted metabolomics is an effective method to comprehensively collect information on all metabolites involved in biological phenomena and to find previously undiscovered ones and their key factors (De Vos et al. 2007). On the other hand, when using untargeted metabolomics in plants, we sometimes deal with unknown metabolites that would not have been detected if we were working with bulk samples.

## SCM in different cell types in the same plant tissue

### Explicit metabolite profiles for various phenotypic and functional differences in the same tissue

Single-cell metabolome analysis in different cell types within the same plant tissue can primarily reveal the presence of cellular heterogeneity. If metabolic phenotypic analysis can be performed at the cellular level as well as at the whole-plant phenotype level, it can be applied to detailed analysis of cellular phenotypes and gene function analysis. Using single-cell analysis to add metabolomic information to the analysis of well-known phenotypes and genes of specific cells is possible. Ebert et al. 2010 combined single-cell sampling techniques with gas chromatograph-time of flight MS (GC-TOF-MS) profiling analysis to obtain metabolome information from cell-specific pools of 200 cells of *A. thaliana* pavement, basal, or trilobular cells and demonstrated quantification of 117 major metabolites along with differences in the metabolic profiles between cell types (Ebert et al. 2010). The paper highlights not only the differences in metabolites between cell types but also the differences in the size of the respective pools by estimating the absolute amounts of metabolites between cell types at single-cell level, which allows a deeper consideration of single cell-specific functions and shows the utility of metabolomics. In parallel, they estimate pool size by monitoring residual recovery losses by dosing internal standards after microsampling, but it has been described that injection of stably labeled internal standards and dyes does not work well, and problems such as incomplete exploitation of intracellular volume make accurate cell volume estimation one of the issues to be carefully considered when conducting SCM. The contents of *A. thaliana* leaf trichomes, basal cells, and pavement cells were aspirated into a microcapillary under a microscope and characterized by ESI-IMS (ion mobility separation)-MS for metabolites (Zhang et al. 2014). In situ metabolic analysis of these *A. thaliana* epidermal cells revealed metabolic differences among the 3 cell types and identified specific metabolites. IMS allowed identification of isobaric ions or ions close to isobaric, e.g. structural isomers, at the single-cell level. In addition, there are examples of high-resolution separation of tomato trichome units by internal electrode capillary pressure probe ESI MS (IEC-PPESI-MS) with much smaller sample volumes, less than 1 pL (Nakashima et al. 2016). In situ single-cell metabolite profiling of stromal and glandular cells, 2 neighboring cell types that make up the tomato trichome unit, has shown significant differences in metabolite composition between the 2 cell types and between different types of trichomes. The analysis between the different trichome types also revealed significant differences in metabolite profiles, especially flavonoids, but it was their capillary-based SCM that allowed them to identify these cell-specific differences.

In one case, different cell types of Arabidopsis root tissue were collected by FACS separation of GFP-labeled cells, and metabolomics analysis of 5 cell types, endodermis, epidermis, columella, cortex, and stele, was carried out (Moussaieff et al. 2013). The cells were analyzed by UPLC-qTOF-MS (ultra-high performance liquid chromatorgraphy-quadruople time-of-flight mass spectrometry) total ion current chromatograms with nontargeted measurement to create a metabolomic profile. PCA analysis revealed that each type was isolated and that the stele cells were particularly well characterized. Thirteen GSLs were identified in this study, but the pattern of accumulation differed among root cells. Although single-cell studies like this one cannot be done on bulk cells, there are also metabolomic analyses that can be done on bulk cells of the whole root, which is also essential information. Correlation with other omics analyses, such as transcriptome analysis, would also support the data. It would be possible to further validate the comparison of metabolome data and omics data, such as transcriptome data at the single-cell level, but the comparison does not seem to be that simple. When sorting cells by FACS or other methods, it must be kept in mind that the cell wall is removed during protoplasting, and this will have an effect, such as on the level of processing, loss of cell wall, and other component information. Acquiring single cells under conditions close to their original location is difficult, but the combination of imaging MS, such as in situ and spectral imaging with Raman microscopy, may break through this problem.

### Estimation of metabolite biosynthesis and flow using SCM and in situ location-based information

Imaging MS (IMS) for one cell in different tissues is useful not only for the transient localization of metabolites but also for estimating their complex biosynthesis, metabolism, and enzyme and transporter functions. The method used to determine the localization of the secondary metabolites, alkaloids, in stem and leaf tissue of the medicinal plant *Catharanthus roseus* is described (Yamamoto et al. 2022). Combining IMS and single-cell MS in *C. roseus* stems involved acquiring and measuring 4 different cell types and revealed that TIAs (terpernoid indole alkaloids) accumulate in idioblast cells and laticifer cells in the stem (Yamamoto et al. 2016). In leaf tissue, the mechanism by which TIAs are biosynthesized from their central precursor, strictosidine, to the various other TIAs in the plant was investigated by imaging MS and live single-cell MS (Yamamoto et al. 2019). Most TIA precursors (iridoid) were found to localize to epidermal cells in leaf tissue, while major TIAs such as serpentine and vindoline were found to localize to idioblast cells instead. They accumulate in both. Bindrin accumulation was also found to be increased in idioblast cells of elongated leaves by analyzing different leaf growth stages. It is possible to know that the accumulation of secondary metabolites in a particular plant is site specific or varies with the growth stage and other factors. In addition, when the target metabolite and its metabolic intermediates are known, the analysis will provide insights into how the metabolism is catalyzed, and it will be possible to investigate the biosynthesis mechanism from there.

Other studies have investigated how specific metabolites are produced and stored. The main constituents of cannabis are cannabinoids, which are produced from stem-like glandular clusters (trichomes) in the female flower. Although it was already known that these trichomes produce resins containing cannabinoids, such as tetrahydrocannabinolic acid and cannabidiolic acid as well as distinct types of secondary metabolites (Tanney et al. 2021), analysis of the single-cell intracellular components of the trichomes using microcapillaries has revealed in detail that the metabolic components vary with flower development and trichome type (Livingston et al. 2020). They have combined analysis of metabolic components and transcriptomes to characterize differences in lipid composition inside the glandular trichomes of cannabis, along with specific fluorescence. They have analyzed microcapillary samples of stem trichomes and sessile early maturing trichomes to determine whether metabolic specialization occurs in cannabis stem trichomes and to explore the gene coexpression network of cannabidiolic acid synthase in cannabinoid biosynthesis and storage. The changes in the components of these trichomes have been analyzed by microcapillary and discussed together with the results of transcriptome analysis. Combined with transcriptome analysis, the identification and characterization of 2 previously unknown, highly expressed monoterpene synthases highlights the metabolic specialization of stem trichomes for monoterpene production.

## SCM in same cell types in the same plant tissue

### Proving the heterogeneity of identical cells in the same tissue

Molecular components of petal pigments of the torenia plant (*Torenia hybrida*) were analyzed on a single-cell basis using a combination of laser microsampling and nanoflow liquid chromatography-electron spray ionization MS (LC-ESIMS). This has been successful in determining the differences in the proportion of anthocyanin molecular components in 3 regions of distinct parts of the petal of the torenia at the single-cell level, as well as measuring the number of anthocyanins in detail (Kajiyama et al. 2006). Although single-cell analysis by capillary can preserve location information, it is difficult to handle many cells, and given the heterogeneity of reactions in the same tissue, a bird's-eye view of the entire space by in situ imaging MS in addition to single-cell metabolite measurements is likely to be necessary. The results of Sugahara et al. 2019 show that the resolution of imaging MS is becoming higher and more quantitative. They have identified novel flavonol 3-O-glycosides other than violanine, flavonol 3-O-glycosides, and violanthin expressed in viola petals, and have investigated whether the flavonoids act as co-pigments of the blue coloration of the petals. To clarify the spatial distribution of the flavonoids, they made a detailed map at the pixel level based on the structure and amount of flavonoid from MALDI TOF-MS imaging measurements. Slight differences in coloration and the amounts of flavonoids comprising them revealed flavonoids that affect the coloration mechanism of viola. They also revealed that excess flavonol 3-O-glycoside molecules surround the violanin quinonoidal form, and it is this which inhibits coloration rather than the pH in the vacuole (Sugahara et al. 2019).

Shrestha et al. demonstrated that cell-by-cell imaging is possible using LAESI in situ without disrupting onion (*Allium cepa*) epidermal cells (Shrestha and Vertes 2009; Shrestha et al. 2011).

Cell-specific quantification of the metabolite cyanidin, the ion responsible for the purple pigment in onion epidermal cells, was performed and found to correlate well with cell color in the tissue. Chemical imaging using single cells as voxels can reflect the spatial distribution of biochemical differences within a tissue without the distortion that results from sampling multiple cells within the laser focus spot. This technique can show that cell populations of the same type of cells in the same tissue are chemically heterogeneous.

In situ metabolite analysis in cells and intracellular organelles can be performed nondestructively using Raman microscopy for component analysis imaging and MALDI MS. We also use Raman laser microscopy for single-cell imaging metabolomics. In addition to being at the single-cell level, it has the advantage of being able to acquire spatial information within the cell. Although it is not as widely used in plants because it is not suitable for autofluorescent or black-colored objects such as chloroplasts, it can be used for highly effective metabolite analysis imaging at the single-cell level by using different experimental materials.

### Knowing the change in the proportion of mixed components that retain the spatial information of the cell

Single-cell metabolite analytical imaging is also useful in studies on the utilization of cell walls formed by multiple components and heterogeneity of the same type of cell. For example, it is well known that the cell walls of lignocellulose materials (LCMs) of herbaceous plants, such as switch grass, are heterogeneous complexes with complex cell walls composed of cellulose, hemicellulose, and lignin and that they are used as biofuels. To utilize them as biofuels, a few steps are necessary, such as disrupting the cell wall structure, reducing the crystallinity of the cellulose, increasing the accessible area, and so on. While these steps have been evaluated in terms of cellulose yield, it would be useful to visualize the molecular properties of LCMs directly, so that it is possible to determine quickly which components and steps of the cell wall have been acted upon. To simultaneously know the location and components that are changing in the cell wall, a combination of spatial correlation Raman scattering and SIMS imaging was used to allow evaluation from the same location and the same sample (Li et al. 2010), and analysis in *Miscanthus* (*Miscanthus* × *giganteus*) which made increased resolution possible (Leng et al. 2022).

## SCM in plant cultured cells in time resolution

SCM corresponding to the progression of life phenomena in the same cell is now also possible, allowing life phenomena to be traced with greater precision. Unlike animal cells, which complete division by centrifugally contracting from the outside to the inside, plant cells complete cell division by centrifugally forming a cell plate as a new cell wall from the center. This phenomenon from the appearance of the cell plate to the completion of insertion is an important structure for plant cells, not only for the distribution of genetic information but also for the subsequent morphogenesis of the cell. Microscopic imaging analysis of this process has revealed that the process of cell plate formation, cytoskeletal dynamics, and cell-to-cell movement of organelles are tightly regulated in parallel with the progress of mitosis (Gunning and Wick 1985; Kutsuna and Hasezawa 2002).

From G2 late metaphase to prophase, a transient band structure of microtubules, the prophase band (PPB), appears (Mineyuki et al. 1988) and leaves several molecular traces; the PPB disappears after prophase but is involved in establishing future mitotic regions (Palevitz and Hepler 1974; Dixit and Cyr 2002; Yoneda et al. 2005; Takeuchi et al. 2016). From PPB degradation in prometaphase to metaphase, microtubules are arranged as mitotic main axes that separate chromosomes between daughter cells (Wick et al. 1981). In anaphase, the cytokinetic apparatus phragmoplast, a complex of cytoskeleton and membrane, assembles at the center of the cell (Liu et al. 2011). Cell plate material is synthesized in the Golgi, packed as vesicles, and transported to the location of the pragmoplast (Otegui and Staehelin 2004). From anaphase to telophase, the phragmoplast spreads toward the cell cortex by centrifugal force and fuses with nearby vesicles. Using the phragmoplast as a scaffold, the cell plate expands and adheres to the cell wall, and the plant cell completes cytokinesis (Smith 2001; Van Damme et al. 2007). The sequence of chromosomes, cytoskeleton, and vesicles moves in a coordinated and undisturbed manner. In order to understand the basic mechanism of this movement, we thought that observing the molecular changes and behavior during cell division would provide important insights. Tobacco cell cultures have been used to profile metabolic components for different periods of intracellular events. Since the M phase of the cell cycle cannot be perfectly synchronized, only single cells of interest were obtained with a nano spray tip. The chromosomes of the visualized cells were monitored under a microscope, and nontargeted measurements were made with Orbitrap LC-MS (Okubo-Kurihara et al. 2022). Changes in metabolic components throughout M phase were previously unknown. However, we determined that the metabolic profile captured significant changes in lipid metabolism during the mitotic subphase, reflecting the appearance and degradation of membrane vesicles involved in cell plate insertion, which had previously been captured by microscopy. Future studies will focus on the involvement of lipids in the cell cycle, such as changes in the progression of cell cycle progression when specific lipids are altered.

## SCM of subcellular compartments in single cell

### Infer movement of substances in and out of the vacuole via transporters

While some metabolomics has been done with vacuoles isolated from bulk-cell protoplasts, there are examples of single-cell organelle metabolomics achieved by utilizing giant internodal cells that are about 20 cm in length. By comparing metabolites between cytoplasm and vacuoles, they have identified vacuole-specific metabolites, providing information on vacuolar transport and metabolic systems. Organelle metabolomics based on single-cell analysis must give more accurate information because the cells from which the isolate is derived and the source of the organelle match. Using *Celtis australis* (*C. australis)* internodes, a single vacuole was obtained and separated into 2 fractions of cytoplasm (Oikawa et al. 2011). Metabolomics of single organelles for these fractions was performed using a metabolomic method combining CE-MS. CE-MS is a useful analytical method for detecting ionic metabolites, such as amino acids, organic acids, and nucleotides, and in this report 125 known metabolites were detected under changing light conditions. The transport of metabolites in and out of the vacuole could be analyzed promptly, providing insight into the function of the vacuole.

### Metabolome profile of lipid droplet

Although lipid metabolites have been analyzed in bulk cells by extracting total lipids from conventional tissues, direct organelle MS (DOMS) is a high-resolution MS profiling method that directly visualizes and extracts lipid droplets (LDs) from plant tissues when the cells are more in their original state and analyzes their lipid composition (Horn et al. 2011; Horn and Chapman 2011, Oikawa et al. 2011). DOMS was performed on LDs isolated from mature cotton embryos and profiled. Variations in triacylglycerol (TAG) composition were verified.

### Metabolome profile of granule

In animal cells, Masujima used a method called LSC-MS to detect 66 granule-specific peaks and 6 cytosol-specific peaks in living cells with great sensitivity by extracting only intracellular granules with a nano spray tip and comparing them with cytosol components (Masujima 2009). This data shows that granules have special components stored in them. By changing the diameter of the spray tip, it will be possible to measure a single lipid body of a plant cell.

Although a single organelle metabolome provides interesting insights, it should not be considered to be the entire information of a single cell, since it may not acquire all that is contained in that cell. In many cases, cells are separated by protoplast treatment or other methods to obtain a single cell, but care must be taken when analyzing the data because the original cellular data may be lost as a result of enzymatic treatment of the cell wall.

## SCM in changes in environment conditions

Metabolites in plants under various biotic and abiotic stresses have been identified and quantified at the single-cell level (Katam et al. 2022). This information provides various clues for understanding plant phenomena and responses against stress. In addition, the identification of biomarkers that have been buried in the bulk of plant metabolome data will be useful for diagnosis of appropriate environmental stresses and for agricultural applications.

Initially, it is necessary to know the relationship between bulk and single-cell samples. In a study in *Vicia fava*, single leaf cells were separated on a nanoelectrophoresis chip under a microscope under various humidity and wound conditions. ABA or JA-Ile was measured by single-cell MS/MS and compared to standard LC-MS/MS for bulk analysis (Shimizu et al. 2015).

Single-cell analysis is also useful in crops. Wada et al. 2020 examined pollen contents and gave deep insights into differences in resistance to heat. Rice seeds from 2 cultivars, a heat-susceptible cultivar and heat-tolerant cultivar, were tested for on-site metabolomics of single pollen grains (Wada et al. 2020). They measured metabolites directly using picoliter pressure-probe-ESI MS. In mature pollen, differences between the 2 cultivars were detected for several metabolites, including phosphatidylinositol (PI) (34:3); more PI content was detected in heat-tolerant pollen, regardless of treatment, than in the heat-susceptible cultivar pollen. PI is a precursor of phosphoinositide, which induces multiple signals involved in pollen germination and tube elongation, suggesting that active synthesis of PI (34:3) prior to germination may be closely related to the maintenance of ear fertilization under elevated temperature. It also shows that metabolites like fatty acids and amino acids can be detected in small sample volumes of about 5 pL.

As previously mentioned, the number of secondary metabolites in plants is estimated to be as high as 200,000 molecular species, and further development of methods for SCM in plants is expected to yield much knowledge about components and their metabolism that have not yet been clarified, such as the production of metabolites in specific cells under different environmental stresses and other conditions.

## Conclusion and future perspectives

Molecules that are present in a small number of cell types, such as metabolites that have been newly identified by single-cell analysis, may be diluted when the entire tissue is assayed, but single-cell analysis can reliably extract these important metabolites. It is also a powerful tool that can precisely predict phenotypic and other information and can be combined with imaging MS to draw out spatial information. The analysis of data will sometimes be difficult, whether the data obtained is the essence of the cell or a fluctuation, but it can be solved by sheer numbers. Combining the methods described here will enable efficient single-cell analysis rather than just gut single-cell isolation. The hierarchy between imaging and metabolomics is close, and the combined imaging mass gives a lot of information about the organism in its native state.

Advances in technology and analytical methods have allowed the comprehensive analysis of cellular metabolism, enabling the discovery of novel metabolic pathways and metabolic diseases. Furthermore, developments in automated high throughput microfluid-based technologies must be combined to the MS aiming for quantitative study to access large cell population in a shorter time without losing cells native environment.

SCM in plants is still in an early stage of development; however, as we establish experimental systems and solve the mysteries of our target biological phenomena, the advantage of being able to analyze what is in individual cells is a motivating factor to overcome any difficulties that arise.

Outstanding questions

- Since single-cell analysis reflects the individuality of each cell, we must consider statistical analysis that does not simply homogenize the data when analyzing them.
- Reagents and devices applicable to animal cells and tissues may not be applicable to plant cells due to the presence of cell walls, or may require the removal of cell walls, or they have not been tested, and methods are needed to overcome these.
- Even if valuable information is extracted from exhaustive data, it is not easy to verify said information because the results are for one single cell.

## References

[kiad357-B1] Ali A , Davidson S, Fraenkel E, Gilmore I, Hankemeier T, Kirwan JA, Lane AN, Lanekoff I, Larion M, McCall LI, et al Single cell metabolism: current and future trends. Metabolomics. 2022:18(10):77. 10.1007/s11306-022-01934-3 36181583 PMC10063251

[kiad357-B2] Altschuler SJ , Wu LF. Cellular heterogeneity: do differences make a difference? Cell 2010:141(4):559–563. 10.1016/j.cell.2010.04.033 20478246 PMC2918286

[kiad357-B3] Autebert J , Coudert B, Bidard FC, Pierga JY, Descroix S, Malaquin L, Viovy JL. Microfluidic: an innovative tool for efficient cell sorting. Methods. 2012:57(3):297–307. 10.1016/j.ymeth.2012.07.002 22796377

[kiad357-B4] Baret JC , Miller OJ, Taly V, Ryckelynck M, El-Harrak A, Frenz L, Rick C, Samuels ML, Hutchison JB, Agresti JJ, et al Fluorescence-activated droplet sorting (FADS): efficient microfluidic cell sorting based on enzymatic activity. Lab Chip. 2009:9(13):1850–1858. 10.1039/b902504a 19532959

[kiad357-B5] Brown VL , Liu Q, He L. Matrix-enhanced surface-assisted laser desorption/ionization mass spectrometry (ME-SALDI-MS) for mass spectrometry imaging of small molecules. Methods Mol Biol. 2015:1203:175–184. 10.1007/978-1-4939-1357-2_17 25361677

[kiad357-B6] Chang Y , Chen X, Zhou Y, Wan J. Deformation-based droplet separation in microfluidics. Ind Eng Chem Res. 2020:59(9):3916–3921. 10.1021/acs.iecr.9b04823

[kiad357-B7] Costa AB , Graham Cooks R. Simulated splashes: elucidating the mechanism of desorption electrospray ionization mass spectrometry. Chem Phys Lett. 2008:464(1-3):1–8. 10.1016/j.cplett.2008.08.020

[kiad357-B8] Deshpande S , Birnie A, Dekker C. On-chip density-based purification of liposomes. Biomicrofluidics. 2017:11(3):034106. 10.1063/1.4983174 PMC5422205 28529672

[kiad357-B9] de Souza LP , Borghi M, Fernie A. Plant single-cell metabolomics-challenges and perspectives. Int J Mol Sci. 2020:21(23):8987. 10.3390/ijms21238987 33256100 PMC7730874

[kiad357-B10] De Vos RC , Moco S, Lommen A, Keurentjes JJ, Bino RJ, Hall RD. Untargeted large-scale plant metabolomics using liquid chromatography coupled to mass spectrometry. Nat Protoc. 2007:2(4):778–791. 10.1038/nprot.2007.95 17446877

[kiad357-B11] Dixit R , Cyr RJ. Spatio-temporal relationship between nuclear-envelope breakdown and preprophase band disappearance in cultured tobacco cells. Protoplasma. 2002:219(1-2):116–121. 10.1007/s007090200012 11926062

[kiad357-B12] Ebert B , Zöller D, Erban A, Fehrle I, Hartmann J, Niehl A, Kopka J, Fisahn J. Metabolic profiling of *Arabidopsis thaliana* epidermal cells. J Exp Bot. 2010:61(5):1321–1335. 10.1093/jxb/erq002 20150518 PMC2837255

[kiad357-B13] Fujii T , Matsuda S, Tejedor ML, Esaki T, Sakane I, Mizuno H, Tsuyama N, Masujima T. Direct metabolomics for plant cells by live single-cell mass spectrometry. Nat Protoc. 2015:10(9):1445–1456. 10.1038/nprot.2015.084 26313480

[kiad357-B14] Gach PC , Shih SC, Sustarich J, Keasling JD, Hillson NJ, Adams PD, Singh AK. A droplet microfluidic platform for automating genetic engineering. ACS Synth Biol. 2016:5(5):426–433. 10.1021/acssynbio.6b00011 26830031

[kiad357-B15] Gao P , Xu G. Mass-spectrometry-based microbial metabolomics: recent developments and applications. Anal Bioanal Chem. 2015:407(3):669–680. 10.1007/s00216-014-8127-7 25216964

[kiad357-B16] Gao SQ , Zhao JH, Guan Y, Tang YS, Li Y, Liu LY. Mass spectrometry imaging technology in metabolomics: a systematic review. Biomed Chromatogr. 2022:37(7):e5494. 10.1002/bmc.5494 36044038

[kiad357-B17] Gomez-Zepeda D , Frausto M, Nájera-González HR, Herrera-Estrella L, Ordaz-Ortiz JJ. Mass spectrometry-based quantification and spatial localization of small organic acid exudates in plant roots under phosphorus deficiency and aluminum toxicity. Plant J. 2021:106(6):1791–1806. 10.1111/tpj.15261 33797826

[kiad357-B18] Grønlund JT , Eyres A, Kumar S, Buchanan-Wollaston V, Gifford ML. Cell specific analysis of Arabidopsis leaves using fluorescence activated cell sorting. J Vis Exp. 2012:4(68):4214. 10.3791/4214 PMC3490320 23070217

[kiad357-B19] Gunning BE , Wick SM. Preprophase bands, phragmoplasts, and spatial control of cytokinesis. J Cell Sci Suppl. 1985:2:157–179. 10.1242/jcs.1985.supplement_2.9 3867671

[kiad357-B20] Guo S , Zhang C, Le A. The limitless applications of single-cell metabolomics. Curr Opin Biotechnol. 2021:71:115–122. 10.1016/j.copbio.2021.07.015 34339935 PMC8592278

[kiad357-B21] Haidas D , Bachler S, Köhler M, Blank LM, Zenobi R, Dittrich PS. Microfluidic platform for multimodal analysis of enzyme secretion in nanoliter droplet arrays. Anal Chem. 2019:91(3):2066–2073. 10.1021/acs.analchem.8b04506 30571917

[kiad357-B22] Hall R , Beale M, Fiehn O, Hardy N, Sumner L, Bino R. Plant metabolomics. Plant Cell. 2002:14(7):1437–1440. 10.1105/tpc.140720 12119365 PMC543394

[kiad357-B23] Hansen RL , Lee YJ. High-spatial resolution mass spectrometry imaging: toward single cell metabolomics in plant tissues. Chem Rec. 2018:18(1):65–77. 10.1002/tcr.201700027 28685965

[kiad357-B24] Harkewicz R , Dennis EA. Applications of mass spectrometry to lipids and membranes. Annu Rev Biochem. 2011:80(1):301–325. 10.1146/annurev-biochem-060409-092612 21469951 PMC3410560

[kiad357-B25] Hazra S , Jayaprakash KS, Pandian K, Raj A, Mitra SK, Sen AK. Non-inertial lift induced migration for label-free sorting of cells in a co-flowing aqueous two-phase system. Analyst. 2019:144(8):2574–2583. 10.1039/C8AN02267D 30821313

[kiad357-B26] Heinemann M , Zenobi R. Single cell metabolomics. Curr Opin Biotechnol. 2011:2(1):26–31. 10.1016/j.copbio.2010.09.008 20934866

[kiad357-B27] Horn PJ , Chapman KD. Organellar lipidomics. Plant Signal Behav. 2011:6(10):1594–1596. 10.4161/psb.6.10.17133 21918374 PMC3256393

[kiad357-B28] Horn PJ , Ledbetter NR, James CN, Hoffman WD, Case CR, Verbeck GF, Chapman KD. Visualization of lipid droplet composition by direct organelle mass spectrometry. J Biol Chem. 2011:286(5):3298–3306. 10.1074/jbc.M110.186353 21118810 PMC3030335

[kiad357-B29] Huebner A , Srisa-Art M, Holt D, Abell C, Hollfelder F, deMello AJ, Edel JB. Quantitative detection of protein expression in single cells using droplet microfluidics. Chem Commun (Camb). 2007:28(12):1218–1220. 10.1039/b618570c 17356761

[kiad357-B30] Huh D , Bahng JH, Ling Y, Wei HH, Kripfgans OD, Fowlkes JB, Grotberg JB, Takayama S. Gravity-driven microfluidic particle sorting device with hydrodynamic separation amplification. Anal Chem. 2007:79(4):1369–1376. 10.1021/ac061542n 17297936 PMC2527745

[kiad357-B31] Jayaprakash KS , Sen AK. Droplet encapsulation of particles in different regimes and sorting of particle-encapsulating-droplets from empty droplets. Biomicrofluidics. 2019:13(3):034108. 10.1063/1.5096937 PMC6517185 31123540

[kiad357-B32] Jindal A , Gupta P, Jayadeva, Sengupta D. Discovery of rare cells from voluminous single cell expression data. Nat Commun. 2018:9(1):4719. 10.1038/s41467-018-07234-6 30413715 PMC6226447

[kiad357-B33] Kajiyama S , Harada K, Fukusaki E, Kobayashi A. Single cell-based analysis of torenia petal pigments by a combination of ArF excimer laser micro sampling and nano-high performance liquid chromatography (HPLC)-mass spectrometry. J Biosci Bioeng. 2006:102(6):575–578. 10.1263/jbb.102.575 17270726

[kiad357-B34] Karthick S , Pradeep PN, Kanchana P, Sen AK. Acoustic impedance-based size-independent isolation of circulating tumour cells from blood using acoustophoresis. Lab Chip. 2018:18(24):3802–3813. 10.1039/C8LC00921J 30402651

[kiad357-B35] Katam R , Lin C, Grant K, Katam CS, Chen S. Advances in plant metabolomics and its applications in stress and single-cell biology. Int J Mol Sci. 2022:23(13):6985. 10.3390/ijms23136985 35805979 PMC9266571

[kiad357-B36] Kempa EE , Smith CA, Li X, Bellina B, Richardson K, Pringle S, Galman JL, Turner NJ, Barran PE. Coupling droplet microfluidics with mass spectrometry for ultrahigh-throughput analysis of complex mixtures up to and above 30 Hz. Anal Chem. 2020:92(18):12605–12612. 10.1021/acs.analchem.0c02632 32786490 PMC8009470

[kiad357-B37] Klein AM , Mazutis L, Akartuna I, Tallapragada N, Veres A, Li V, Peshkin L, Weitz DA, Kirschner MW. Droplet barcoding for single-cell transcriptomics applied to embryonic stem cells. Cell. 2015:161(5):1187–1201. 10.1016/j.cell.2015.04.044 26000487 PMC4441768

[kiad357-B38] Koroleva OA , Farrar JF, Deri Tomos A, Pollock CJ. Carbohydrates in individual cells of epidermis, mesophyll, and bundle sheath in barley leaves with changed export or photosynthetic rate. Plant Physiol. 1998:118(4):1525–1532. 10.1104/pp.118.4.1525 9847129 PMC34771

[kiad357-B39] Koroleva OA , Farrar JF, Tomos AD, Pollock CJ. Patterns of solute in individual mesophyll, bundle sheath and epidermal cells of barley leaves induced to accumulate carbohydrate. New Phytologist. 1997:136(1):97–104. 10.1111/j.1469-8137.1997.tb04735.x

[kiad357-B40] Korte AR , Yandeau-Nelson MD, Nikolau BJ, Lee YJ. Subcellular-level resolution MALDI-MS imaging of maize leaf metabolites by MALDI-linear ion trap-orbitrap mass spectrometer. Anal Bioanal Chem. 2015:407(8):2301–2309. 10.1007/s00216-015-8460-5 25618761

[kiad357-B41] Kutsuna N , Hasezawa S. Dynamic organization of vacuolar and microtubule structures during cell cycle progression in synchronized tobacco BY-2 cells. Plant Cell Physiol. 2002:43(9):965–973. 10.1093/pcp/pcf138 12354913

[kiad357-B42] Lanekoff I , Sharma VV, Marques C. Single-cell metabolomics: where are we and where are we going? Curr Opin Biotechnol. 2022:75:102693. 10.1016/j.copbio.2022.102693 35151979

[kiad357-B43] Leng W , He S, Lu B, Thirumalai RVKG, Nayanathara RMO, Shi J, Zhang R, Zhang X. Raman imaging: an indispensable technique to comprehend the functionalization of lignocellulosic material. Int J Biol Macromol. 2022:220:159–174. 10.1016/j.ijbiomac.2022.08.084 35981669

[kiad357-B44] Li B , Bhandari DR, Janfelt C, Römpp A, Spengler B. Natural products in *Glycyrrhiza glabra* (licorice) rhizome imaged at the cellular level by atmospheric pressure matrix-assisted laser desorption/ionization tandem mass spectrometry imaging. Plant J. 2014:80(1):161–171. 10.1111/tpj.12608 25040821

[kiad357-B46] Li M , Liu H, Zhuang S, Goda K. Droplet flow cytometry for single-cell analysis. RSC Adv. 2021:11(34):20944–20960. 10.1039/D1RA02636D 35479393 PMC9034116

[kiad357-B47] Li M , van Zee M, Goda K, Di Carlo D. Size-based sorting of hydrogel droplets using inertial microfluidics. Lab Chip. 2018:18(17):2575–2582. 10.1039/C8LC00568K 30046787

[kiad357-B45] Li Z , Chu LQ, Sweedler JV, Bohn PW. Spatial correlation of confocal Raman scattering and secondary ion mass spectrometric molecular images of lignocellulosic materials. Anal Chem. 2010:82(7):2608–2611. 10.1021/ac100026r 20205411

[kiad357-B48] Li Z , Wang Z, Pan J, Ma X, Zhang W, Ouyang Z. Single-cell mass spectrometry analysis of metabolites facilitated by cell electro-migration and electroporation. Anal Chem. 2020:92(14):10138–10144. 10.1021/acs.analchem.0c02147 32568528

[kiad357-B49] Lisec J , Schauer N, Kopka J, Willmitzer L, Fernie AR. Gas chromatography mass spectrometry-based metabolite profiling in plants. Nat Protoc. 2006:1(1):387–396. 10.1038/nprot.2006.59 17406261

[kiad357-B50] Liu B , Ho CM, Lee YR. Microtubule reorganization during mitosis and cytokinesis: lessons learned from developing microgametophytes in *Arabidopsis thaliana*. Front Plant Sci. 2011:2:27. 10.3389/fpls.2011.00027 22639587 PMC3355579

[kiad357-B51] Liu R , Pan N, Zhu Y, Yang Z. T-probe: an integrated microscale device for online in situ single cell analysis and metabolic profiling using mass spectrometry. Anal Chem. 2018:90(18):11078–11085. 10.1021/acs.analchem.8b02927 30119596 PMC6583895

[kiad357-B52] Livingston SJ , Quilichini TD, Booth JK, Wong DCJ, Rensing KH, Laflamme-Yonkman J, Castellarin SD, Bohlmann J, Page JE, Samuels AL. Cannabis glandular trichomes alter morphology and metabolite content during flower maturation. Plant J. 2020:101(1):37–56. 10.1111/tpj.14516 31469934

[kiad357-B53] Lorenzo Tejedor M , Mizuno H, Tsuyama N, Harada T, Masujima T. Direct single-cell molecular analysis of plant tissues by video mass spectrometry. Anal Sci. 2009:25(9):1053–1055. 10.2116/analsci.25.1053 19745529

[kiad357-B54] Lorenzo Tejedor M , Mizuno H, Tsuyama N, Harada T, Masujima T. In situ molecular analysis of plant tissues by live single-cell mass spectrometry. Anal Chem. 2012:84(12):5221–5228. 10.1021/ac202447t 22243623

[kiad357-B55] Macosko EZ , Basu A, Satija R, Nemesh J, Shekhar K, Goldman M, Tirosh I, Bialas AR, Kamitaki N, Martersteck EM, et al Highly parallel genome-wide expression profiling of individual cells using nanoliter droplets. Cell. 2015:161(5):1202–1214. 10.1016/j.cell.2015.05.002 26000488 PMC4481139

[kiad357-B56] Malone M , Leigh RA, Tomos AD. Extraction and analysis of sap from individual wheat leaf cells: the effect of sampling speed on the osmotic pressure of extracted sap. Plant Cell Environ. 1989:12(9):919–926. 10.1111/j.1365-3040.1989.tb01971.x

[kiad357-B57] Martin K , Henkel T, Baier V, Grodrian A, Schön T, Roth M, Michael Köhler J, Metze J. Generation of larger numbers of separated microbial populations by cultivation in segmented-flow microdevices. Lab Chip. 2003:3(3):202–207. 10.1039/B301258C 15100775

[kiad357-B58] Masuda K , Abouleila Y, Ali A, Yanagida T, Masujima T. Live single-cell mass spectrometry (LSC-MS) for plant metabolomics. Methods Mol Biol. 2018:1778:269–282. 10.1007/978-1-4939-7819-9_19 29761445

[kiad357-B59] Masujima T . Live single-cell mass spectrometry. Anal Sci. 2009:25(8):953–960. 10.2116/analsci.25.953 19667470

[kiad357-B60] Mazutis L , Gilbert J, Ung WL, Weitz DA, Griffiths AD, Heyman JA. Single-cell analysis and sorting using droplet-based microfluidics. Nat Protoc. 2013:8(5):870–891. 10.1038/nprot.2013.046 23558786 PMC4128248

[kiad357-B61] Mineyuki Y , Wick SM, Gunning BE. Preprophase bands of microtubules and the cell cycle: kinetics and experimental uncoupling of their formation from the nuclear cycle in onion root-tip cells. Planta. 1988:174(4):518–526. 10.1007/BF00634482 24221569

[kiad357-B62] Misra BB , Assmann SM, Chen S. Plant single-cell and single-cell-type metabolomics. Trends Plant Sci. 2014:19(10):637–646. 10.1016/j.tplants.2014.05.005 24946988

[kiad357-B63] Moussaieff A , Rogachev I, Brodsky L, Malitsky S, Toal TW, Belcher H, Yativ M, Brady SM, Benfey PN, Aharoni A. High-resolution metabolic mapping of cell types in plant roots. Proc Natl Acad Sci U S A. 2013:110(13):E1232–E1241. 10.1073/pnas.1302019110 23476065 PMC3612672

[kiad357-B64] Müller T , Oradu S, Ifa DR, Cooks RG, Kräutler B. Direct plant tissue analysis and imprint imaging by desorption electrospray ionization mass spectrometry. Anal Chem. 2011:83(14):5754–5761. 10.1021/ac201123t 21675752 PMC3137229

[kiad357-B65] Nakashima T , Wada H, Morita S, Erra-Balsells R, Hiraoka K, Nonami H. Single-cell metabolite profiling of stalk and glandular cells of intact trichomes with internal electrode capillary pressure probe electrospray ionization mass spectrometry. Anal Chem. 2016:88(6):3049–3057. 10.1021/acs.analchem.5b03366 26845634

[kiad357-B66] Nawaz AA , Chen Y, Nama N, Nissly RH, Ren L, Ozcelik A, Wang L, McCoy JP, Levine SJ, Huang TJ. Acoustofluidic fluorescence activated cell sorter. Anal Chem. 2015:87(24):12051–12058. 10.1021/acs.analchem.5b02398 26331909 PMC4888785

[kiad357-B67] Newell CL , Vorng JL, MacRae JI, Gilmore IS, Gould AP. Cryogenic OrbiSIMS localizes semi-volatile molecules in biological tissues. Angew Chem Int Ed Engl. 2020:59(41):18194–18200. 10.1002/anie.202006881 32603009 PMC7589292

[kiad357-B68] Oikawa A , Matsuda F, Kikuyama M, Mimura T, Saito K. Metabolomics of a single vacuole reveals metabolic dynamism in an alga *Chara australis*. Plant Physiol. 2011:157(2):544–551. 10.1104/pp.111.183772 21846815 PMC3192564

[kiad357-B69] Oikawa A , Saito K. Metabolite analyses of single cells. Plant J. 2012:70(1):30–38. 10.1111/j.1365-313X.2012.04967.x 22449041

[kiad357-B70] Okubo-Kurihara E , Ali A, Hiramoto M, Kurihara Y, Abouleila Y, Abdelazem EM, Kawai T, Makita Y, Kawashima M, Esaki T, et al Tracking metabolites at single-cell resolution reveals metabolic dynamics during plant mitosis. Plant Physiol. 2022:189(2):459–464. 10.1093/plphys/kiac093 35301535 PMC9157120

[kiad357-B71] Olefirowicz TM , Ewing AG. Capillary electrophoresis for sampling single nerve cells. Chimia (Aarau). 1991:45(4):106–106. 10.2533/chimia.1991.106

[kiad357-B72] Ortiz-Ramírez C , Arevalo ED, Xu X, Jackson DP, Birnbaum KD. An efficient cell sorting protocol for maize protoplasts. Curr Protoc Plant Biol. 2018:3(3):e20072. 10.1002/cppb.20072 PMC6135669 30138552

[kiad357-B73] Otegui MS , Staehelin LA. Electron tomographic analysis of post-meiotic cytokinesis during pollen development in *Arabidopsis thaliana*. Planta. 2004:218(4):501–515. 10.1007/s00425-003-1125-1 14610676

[kiad357-B74] Palevitz BA , Hepler PK. The control of the plane of division during stomatal differentiation in allium. Chromosoma. 1974:46(3):297–326. 10.1007/BF00284884

[kiad357-B75] Pan N , Rao W, Kothapalli NR, Liu R, Burgett AW, Yang Z. The single-probe: a miniaturized multifunctional device for single cell mass spectrometry analysis. Anal Chem. 2014:86(19):9376–9380. 10.1021/ac5029038 25222919

[kiad357-B76] Passarelli MK , Ewing AG. Single-cell imaging mass spectrometry. Curr Opin Chem Biol. 2013:17(5):854–859. 10.1016/j.cbpa.2013.07.017 23948695 PMC3823831

[kiad357-B77] Patel MK , Pandey S, Kumar M, Haque MI, Pal S, Yadav NS. Plants metabolome study: emerging tools and techniques. Plants (Basel). 2021:10(11):2409. 10.3390/plants10112409 34834772 PMC8621461

[kiad357-B78] Saito K , Hirai MY, Yonekura-Sakakibara K. Decoding genes with coexpression networks and metabolomics - ‘majority report by precogs’. Trends Plant Sci. 2008:13(1):36–43. 10.1016/j.tplants.2007.10.006 18160330

[kiad357-B79] Sajeesh P , Doble M, Sen AK. Hydrodynamic resistance and mobility of deformable objects in microfluidic channels. Biomicrofluidics. 2014:8(5):054112. 10.1063/1.4897332 PMC4222326 25538806

[kiad357-B80] Samarah LZ , Khattar R, Tran TH, Stopka SA, Brantner CA, Parlanti P, Veličković D, Shaw JB, Agtuca BJ, Stacey G, et al Single-cell metabolic profiling: metabolite formulas from isotopic fine structures in heterogeneous plant cell populations. Anal Chem. 2020:92(10):7289–7298. 10.1021/acs.analchem.0c00936 32314907

[kiad357-B81] Sarabia LD , Boughton BA, Rupasinghe T, van de Meene AML, Callahan DL, Hill CB, Roessner U. High-mass-resolution MALDI mass spectrometry imaging reveals detailed spatial distribution of metabolites and lipids in roots of barley seedlings in response to salinity stress. Metabolomics. 2018:14(5):63. 10.1007/s11306-018-1359-3 29681790 PMC5907631

[kiad357-B82] Satterlee JW , Scanlon MJ. Protoplast isolation from undifferentiated maize seedling shoot tissue. Methods Mol Biol. 2022:2464:123–130. 10.1007/978-1-0716-2164-6_9 35258829

[kiad357-B83] Schauer N , Fernie AR. Plant metabolomics: towards biological function and mechanism. Trends Plant Sci. 2006:11(10):508–516. 10.1016/j.tplants.2006.08.007 16949327

[kiad357-B84] Senoner M , Unger WES. SIMS Imaging of the nanoworld: applications in science and technology. J Anal At Spectrom. 2012:27(7):1050–1068. 10.1039/c2ja30015j

[kiad357-B85] Sheen J . Signal transduction in maize and Arabidopsis mesophyll protoplasts. Plant Physiol. 2001:127(4):1466–1475. 10.1104/pp.010820 11743090 PMC1540179

[kiad357-B86] Shimizu T , Miyakawa S, Esaki T, Mizuno H, Masujima T, Koshiba T, Seo M. Live single-cell plant hormone analysis by video-mass spectrometry. Plant Cell Physiol. 2015:56(7):1287–1296. 10.1093/pcp/pcv042 25759328

[kiad357-B87] Shrestha B , Patt JM, Vertes A. In situ cell-by-cell imaging and analysis of small cell populations by mass spectrometry. Anal Chem. 2011:83(8):2947–2955. 10.1021/ac102958x 21388149

[kiad357-B88] Shrestha B , Vertes A. In situ metabolic profiling of single cells by laser ablation electrospray ionization mass spectrometry. Anal Chem. 2009:81(20):8265–8271. 10.1021/ac901525g 19824712

[kiad357-B90] Smith CA , Li X, Mize TH, Sharpe TD, Graziani EI, Abell C, Huck WT. Sensitive, high throughput detection of proteins in individual, surfactant-stabilized picoliter droplets using nanoelectrospray ionization mass spectrometry. Anal Chem. 2013:85(8):3812–3816. 10.1021/ac400453t 23514243

[kiad357-B89] Smith LG . Plant cell division: building walls in the right places. Nat Rev Mol Cell Biol. 2001:2(1):33–39. 10.1038/35048050 11413463

[kiad357-B91] Sparks EE , Benfey PN. Tissue-specific transcriptome profiling in Arabidopsis roots. Methods Mol Biol. 2017:1610:107–122. 10.1007/978-1-4939-7003-2_8 28439860

[kiad357-B92] Sugahara K , Kitao K, Watanabe T, Yamagaki T. Imaging mass spectrometry analysis of flavonoids in blue viola petals and their enclosure effects on violanin during color expression. Anal Chem. 2019:91(1):896–902. 10.1021/acs.analchem.8b03815 30521315

[kiad357-B93] Takahashi K , Kozuka T, Anegawa A, Nagatani A, Mimura T. Development and application of a high-resolution imaging mass spectrometer for the study of plant tissues. Plant Cell Physiol. 2015:56(7):1329–1338. 10.1093/pcp/pcv083 26063395

[kiad357-B94] Takeuchi M , Karahara I, Kajimura N, Takaoka A, Murata K, Misaki K, Yonemura S, Staehelin LA, Mineyuki Y. Single microfilaments mediate the early steps of microtubule bundling during preprophase band formation in onion cotyledon epidermal cells. Mol Biol Cell. 2016:27(11):1809–1820. 10.1091/mbc.e15-12-0820 27053663 PMC4884071

[kiad357-B95] Tan YC , Ho YL, Lee AP. Microfluidic sorting of droplets by size. Microfluid Nanofluid. 2008:4(4):343–348. 10.1007/s10404-007-0184-1

[kiad357-B96] Tanney CAS , Backer R, Geitmann A, Smith DL. Cannabis glandular trichomes: a cellular metabolite factory. Front Plant Sci. 2021:12:721986. 10.3389/fpls.2021.721986 PMC8488169 34616415

[kiad357-B97] Taylor MJ , Mattson S, Liyu A, Stopka SA, Ibrahim YM, Vertes A, Anderton CR. Optical microscopy-guided laser ablation electrospray ionization ion mobility mass spectrometry: ambient single cell metabolomics with increased confidence in molecular identification. Metabolites. 2021:11(4):200. 10.3390/metabo11040200 33801673 PMC8065410

[kiad357-B98] Teh SY , Lin R, Hung LH, Lee AP. Droplet microfluidics. Lab Chip. 2008:8(2):198–220. 10.1039/b715524g 18231657

[kiad357-B99] Terekhov SS , Smirnov IV, Stepanova AV, Bobik TV, Mokrushina YA, Ponomarenko NA, Jr BA, Rubtsova MP, Kartseva OV, Gomzikova MO, et al Microfluidic droplet platform for ultrahigh-throughput single-cell screening of biodiversity. Proc Natl Acad Sci U S A. 2017:114(10):2550–2555. 10.1073/pnas.1621226114 28202731 PMC5347554

[kiad357-B100] Utharala R , Tseng Q, Furlong EEM, Merten CA. A versatile, low-cost, multiway microfluidic sorter for droplets, cells, and embryos. Anal Chem. 2018:90(10):5982–5988. 10.1021/acs.analchem.7b04689 29688703

[kiad357-B101] Van Damme D , Vanstraelen M, Geelen D. Cortical division zone establishment in plant cells. Trends Plant Sci. 2007:12(10):458–464. 10.1016/j.tplants.2007.08.011 17765597

[kiad357-B102] Wada H , Hatakeyama Y, Nakashima T, Nonami H, Erra-Balsells R, Hakata M, Nakata K, Hiraoka K, Onda Y, Nakano H. On-site single pollen metabolomics reveals varietal differences in phosphatidylinositol synthesis under heat stress conditions in rice. Sci Rep. 2020:10(1):2013. 10.1038/s41598-020-58869-9 32029818 PMC7005239

[kiad357-B103] Wang BL , Ghaderi A, Zhou H, Agresti J, Weitz DA, Fink GR, Stephanopoulos G. Microfluidic high-throughput culturing of single cells for selection based on extracellular metabolite production or consumption. Nat Biotechnol. 2014:32(5):473–478. 10.1038/nbt.2857 24705516 PMC4412259

[kiad357-B104] Warnasooriya SN , Montgomery BL. Investigating tissue- and organ-specific phytochrome responses using FACS-assisted cell-type specific expression profiling in *Arabidopsis thaliana*. J Vis Exp. 2010:29(39):1925. 10.3791/1925 PMC3152858 20517200

[kiad357-B105] Wick SM , Seagull RW, Osborn M, Weber K, Gunning BE. Immunofluorescence microscopy of organized microtubule arrays in structurally stabilized meristematic plant cells. J Cell Biol. 1981:89(3):685–690. 10.1083/jcb.89.3.685 7019218 PMC2111797

[kiad357-B106] Wu M , Ozcelik A, Rufo J, Wang Z, Fang R, Jun Huang T. Acoustofluidic separation of cells and particles. Microsyst Nanoeng. 2019:5:32. 10.1038/s41378-019-0064-3 31231539 PMC6545324

[kiad357-B107] Xiong C , Zhou X, He Q, Huang X, Wang J, Peng WP, Chang HC, Nie Z. Development of visible-wavelength MALDI cell mass spectrometry for high-efficiency single-cell analysis. Anal Chem. 2016:88(23):11913–11918. 10.1021/acs.analchem.6b03789 27780355

[kiad357-B108] Yamamoto K , Takahashi K, Caputi L, Mizuno H, Rodriguez-Lopez CE, Iwasaki T, Ishizaki K, Fukaki H, Ohnishi M, Yamazaki M, et al The complexity of intercellular localisation of alkaloids revealed by single-cell metabolomics. New Phytol. 2019:224(2):848–859. 10.1111/nph.16138 31436868

[kiad357-B109] Yamamoto K , Takahashi K, Mizuno H, Anegawa A, Ishizaki K, Fukaki H, Ohnishi M, Yamazaki M, Masujima T, Mimura T. Cell-specific localization of alkaloids in *Catharanthus roseus* stem tissue measured with imaging MS and Single-cell MS. Proc Natl Acad Sci U S A. 2016:113(14):3891–3896. 10.1073/pnas.1521959113 27001858 PMC4833245

[kiad357-B110] Yamamoto K , Takahashi K, O’Connor SE, Mimura T. Imaging MS analysis in *Catharanthus roseus*. Methods Mol Biol. 2022:2505:33–43. 10.1007/978-1-0716-2349-7_2 35732934

[kiad357-B111] Yoneda A , Akatsuka M, Hoshino H, Kumagai F, Hasezawa S. Decision of spindle poles and division plane by double preprophase bands in a BY-2 cell line expressing GFP-tubulin. Plant Cell Physiol. 2005:46(3):531–538. 10.1093/pcp/pci055 15695445

[kiad357-B112] Yu Z , Boehm CR, Hibberd JM, Abell C, Haseloff J, Burgess SJ, Reyna-Llorens I. Droplet-based microfluidic analysis and screening of single plant cells. PLoS One. 2018:13(5):e0196810. 10.1371/journal.pone.0196810 PMC5933695 29723275

[kiad357-B113] Zenobi R . Single-cell metabolomics: analytical and biological perspectives. Science. 2013:342(6163):1243259. 10.1126/science.1243259 24311695

[kiad357-B114] Zhang L , Foreman DP, Grant PA, Shrestha B, Moody SA, Villiers F, Kwak JM, Vertes A. In situ metabolic analysis of single plant cells by capillary microsampling and electrospray ionization mass spectrometry with ion mobility separation. Analyst. 2014:139(20):5079–5085. 10.1039/C4AN01018C 25109271

[kiad357-B115] Zhou Y , Ma Z, Ai Y. Hybrid microfluidic sorting of rare cells based on high throughput inertial focusing and high accuracy acoustic manipulation. RSC Adv. 2019:9(53):31186–31195. 10.1039/C9RA01792E 35529382 PMC9072550

[kiad357-B117] Zhu H , Zou G, Wang N, Zhuang M, Xiong W, Huang G. Single-neuron identification of chemical constituents, physiological changes, and metabolism using mass spectrometry. Proc Natl Acad Sci U S A. 2017:114(10):2586–2591. 10.1073/pnas.1615557114 28223513 PMC5347563

[kiad357-B116] Zhu Y , Liu R, Yang Z. Redesigning the T-probe for mass spectrometry analysis of online lysis of non-adherent single cells. Anal Chim Acta. 2019:1084:53–59. 10.1016/j.aca.2019.07.059 31519234 PMC6746249

